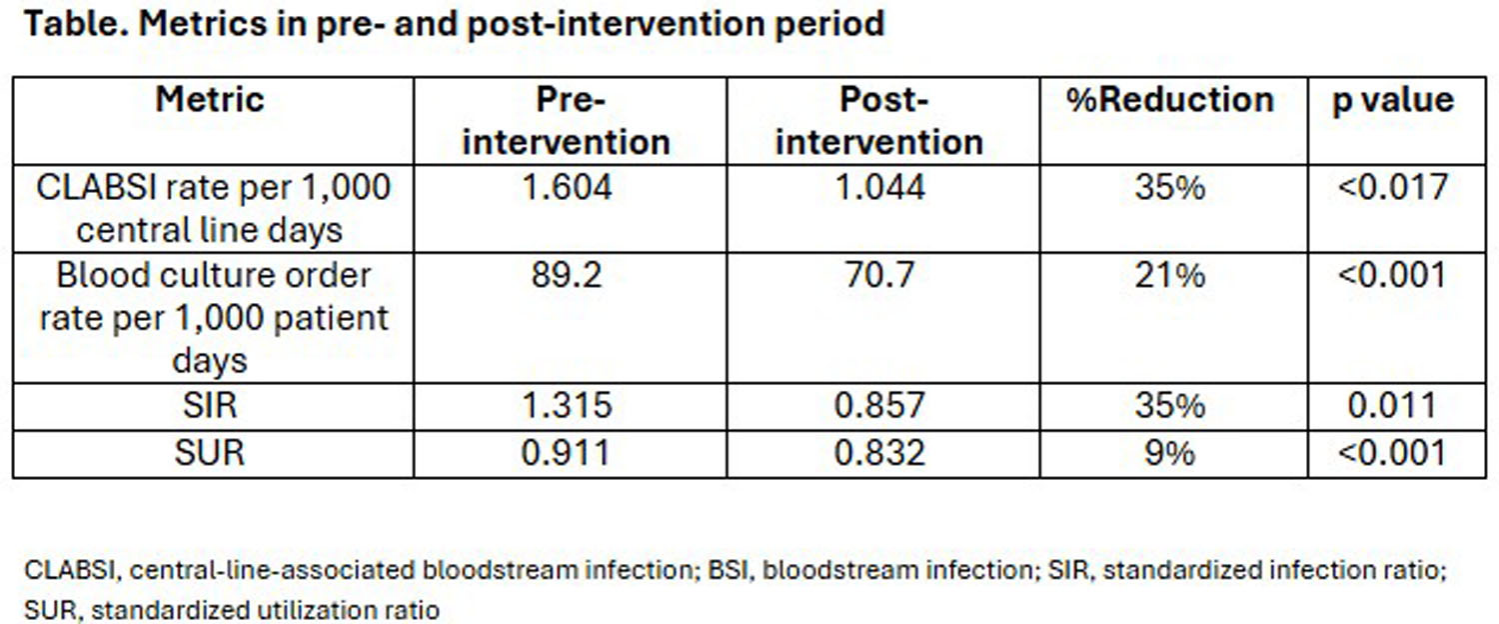# 169 Pathogen Genomic Surveillance in Healthcare: Emerging Ethical Dilemmas in Outbreak Detection and Patient Notification

**DOI:** 10.1017/ash.2026.10570

**Published:** 2026-06-23

**Authors:** Anita Shallal, Jennifer McLenon, Abigail Ruby, Eman Chami, Arielle Gupta, Geehan Suleyman

**Affiliations:** 1 Henry Ford Hospital; 2 Henry Ford Health; 3 Henry Ford Health System

## Abstract

**Background:** Central-line-associated bloodstream infections (CLABSI) are a costly yet preventable healthcare-associated infections, contributing to increased morbidity and mortality. We describe the impact of a multidisciplinary multi-faceted approach on CLABSI rates at our institution. **Methods:** This is a pre—post quasi-experimental retrospective study across an 877-bed, quaternary care academic hospital in Southeast Michigan. The CLABSI rate per 1,000 central-line days, blood culture (BC) order rate per 1,000 patient days, standardized infection ratio (SIR) and standardized utilization ratio (SUR) in the pre-intervention period (1/2023-6/2024) to the post-intervention period (9/2024-8/2025) were compared. CLABSI was determined using National Healthcare Safety Network criteria. The multi-faceted intervention comprised of: (1) implementation of a diagnostic stewardship guideline with an algorithm-based approach to improve appropriate blood-culture ordering; (2) prompt notification of CLABSI-eligible patients to facilitate early evaluation for secondary sources; and (3) use of an electronic central line indications checklist to promote early removal of unnecessary lines, supported by an interprofessional stakeholder meeting in June 2024 that designated unit-level champions for execution and feedback. **Results:** The CLABSI rate significantly decreased from 1.604 to 1.044, resulting in a 35% reduction (p<0.017) [Table]. The SIR significantly decreased from 1.315 to 0.857, a 35% reduction (p=0.011). The SUR significantly decreased from 0.911 to 0.832, a 9% reduction (p<0.001). The BC order rate per 1,000 patient days decreased from 89.2 in the pre-intervention period to 70.7 post-intervention, a 21% reduction (p<0.001). Discussion: A significant reduction in CLABSI rates, central-line utilization, and blood-culture orders was observed following implementation of a multi-faceted approach supported by multidisciplinary collaboration. These findings demonstrate that a coordinated bundled strategy, executed in close collaboration with primary clinical teams, can significantly reduce CLABSI burden in a large academic medical center.

**Figure:**